# Incidence and outcomes of long QTc in acute medical admissions

**DOI:** 10.1111/ijcp.13250

**Published:** 2018-09-17

**Authors:** Rahel Mahmud, Adam Gray, Adam Nabeebaccus, Martin Brunel Whyte

**Affiliations:** ^1^ Department of Acute Medicine King's College Hospital NHS Foundation Trust London UK; ^2^ Department of Cardiology King's College Hospital NHS Foundation Trust London UK; ^3^ Department of Clinical & Experimental Medicine University of Surrey Guildford UK

## Abstract

**Aims:**

Prolonged QT interval on electrocardiogram (ECG) increases the risk of ventricular arrhythmia. Patients admitted to acute medical units (AMU) may be at risk of QT prolongation from multiple, recognised risk factors. Few data exist regarding incidence or outcomes of QT prolongation in acute general medical admissions.

The aims were to determine the incidence of Bazett's‐corrected QT (QTc) prolongation upon admission to AMU; the relationship between QTc and inpatient mortality, length of stay and readmission; proportion with prolonged QTc subsequently administered QT interval‐prolonging drugs.

**Methods:**

Retrospective, observational study of 1000 consecutive patients admitted to an AMU in a large urban hospital. Exclusion criteria: age <18 years, ventricular pacing, poor quality/absent ECG **.** QTc determined manually from ECG obtained within 4‐hours of admission. QTc prolongation considered ≥470 milliseconds (males) and ≥480 milliseconds (females). In both genders, >500 milliseconds was considered severe. Study end‐points, (a) incidence of QTc prolongation at admission; (b) inpatient mortality, length of stay and readmission rates; (c) proportion with QTc prolongation subsequently administered QT interval‐prolonging drugs.

**Results:**

Of 1000 patients, 288 patients were excluded, therefore final sample was n = 712. Patient age (mean ± SD) was 63.1 ± 19.4 years; females 49%. QTc prolongation was present in n = 50 (7%) at admission; 1.7% had QTc interval >500 ms. Of the 50 patients admitted with prolonged QTc, 6 (12%) were subsequently administered QT interval‐prolonging drugs. QTc prolongation was not associated with worse inpatient mortality or readmission rate. Length of stay was greater in those with prolonged QTc, 7.2 (IQR 2.4‐13.2) days vs 3.3 (IQR 1.3‐10.0; *P *=* *0.004), however, in a regression model, presence of QTc did not independently affect length of stay.

**Conclusions:**

QTc interval prolongation is frequent among patients admitted to AMU. QT interval‐prolonging drugs are commonly prescribed to patients presenting with prolonged QTc but whether this affects clinical outcomes is uncertain.

**What's known**

There is a high prevalence and poor outcomes of prolonged QTc in cardiac and cerebrovascular disease. Bazett formula is considered the gold‐standard means to control QT interval for heart rate.

**What's new**

The incidence of prolonged QTc interval (using Bazett formula) in unselected medical admissions is 7%. Automatic QTc measurement records a significantly greater proportion of patients with long QT, whereas Fridericia formula identified fewer patients with prolonged QTc. Lower plasma potassium, even within the normal range, predisposes to prolonged QTc. Patients with long QTc have a longer length of hospital admission.

## 1 INTRODUCTION

Long QTc on electrocardiogram (ECG) can lead to torsade de pointes (TdP). Although this is usually self‐limiting, it may degenerate into ventricular fibrillation. Prevention or treatment of the factors underlying long QT would be expected to have significant clinical benefit.

Hospitalised patients may be at greater risk of long QT than the general population.^1^ Acute medical admissions tend to be elderly and so may be more vulnerable for drug‐induced QTc prolongation and thence arrhythmia, as the QTc interval is known to increase with age.^2^, ^3^ In addition, elderly patients may have other risk factors for QTc interval prolongation, such as the presence of diabetes mellitus, polypharmacy or electrolyte disturbances.^4^

QT prolongation has been shown to be present in up to 45% of patients after cerebrovascular accident (CVA)^5^ and in 18% of patients on a coronary care unit (CCU)^6^ but a much lower prevalence (0.9%) was recorded in a single centre study across all inpatients of a general hospital.^7^ The incidence and prevalence of prolonged QTc in unselected medical admissions to an acute medical unit (AMU) is unknown and the frequency with which QT interval‐prolonging drugs are prescribed to patients at admission and following admission, in this environment, are also unknown. We hypothesised that QT prolongation would be more frequently encountered in acute medical admissions than in a hospital‐wide environment,^7^ thereby posing a considerable clinical risk.

The objectives of this study were to (a) determine the incidence of long QT upon admission to a large urban teaching hospital with an acute medical illness; and (b) determine the proportion of patients receiving QT‐prolonging drugs at the time of admission and the change in prescribing of QT‐prolonging drugs after admission.

## 2 METHODS

A retrospective study of 1000 consecutive patients admitted to an AMU at a 950‐bed, urban teaching hospital. At our institution, ST‐elevation myocardial infarction and acute CVA are triaged directly to the cardiac and stroke services, respectively, and so are not referred to the AMU. Patients routinely have a 12‐lead ECG within 12 hours of admission (Welch‐Allyn^®^). The ECG trace was accessed using EDMonline^®^ software. The first satisfactory ECG obtained for each ED visit was included. If the ECG was noisy, obscuring the relevant onset or offsets, incomplete with some leads missing, or rapid with overlap of the terminal T wave and the next cycle P or QRS waves, then the next ECG obtained during the same ED visit was assessed. If there was no satisfactory ECG, then the case was excluded. Other exclusions were patients aged less than 18 years of age, paced ECG, missing patient ECG or missing patients’ identifiers.

The QT interval represents the time from the beginning of ventricular depolarisation to completion of repolarisation. However, because the QT interval encompasses ventricular depolarisation adjustment of QTc in those with left bundle branch block was made using the formula: measured QT interval minus 50% of LBBB duration.^8^, ^9^

The presence and severity of candidate risk factors (determined from the published literature) were recorded (obtained from the EPR system). These fell under three domains: basic demographic (age, gender^10^, ^11^), biochemistry (capillary glucose at the time of the ECG,^12^ potassium,^13^ calcium and magnesium concentrations^10^, ^14^ obtained closest temporally to ECG acquisition within 12 hours) and medications prescribed within 7‐days of the index ECG. Community‐issued medications are obtained by pharmacist reconciliation with primary care. All data were anonymised. A medication was classified as QTc‐prolonging based upon listing at a pharmacy‐reference website (http://www.crediblemeds.org).^15^ In this listing, medications are sub‐classified as ‘known’, probable’ or ‘conditional’ for QTc prolongation, or whether they are at risk of inducing QTc prolongation in those with congenital long‐QT syndrome.

The principal outcome measure was the incidence of long QTc using Bazett formula in acute medical admissions. Bazett formula is QTc = QT*(HR/60)^1/2^.^16^

Secondary outcome measures were: proportion of patients with prolonged QTc who were subsequently administered drugs known to prolong QTc during their inpatient stay; length of admission; in‐hospital mortality; rate of readmission at 1, 3 and 12 months; inter‐observer agreement (kappa statistic) of QTc length. We also compared the incidence of QT based upon the automatic ECG output, which uses the Bazett formula by default and with the Fridericia formula (QTcF), for which the formula is QTc = QT*(HR/60)^1/3^. QTcF is thought to reflect a more accurate correction factor than Bazett's formula in subjects with faster heart rates.^17^

QT intervals were determined manually by two investigators. QT intervals were determined manually from lead II of 12‐lead ECGs. QT intervals were measured from the earliest QRS deflection to the end of the T wave. The intersection of the down slop of the T wave with the baseline was taken as the end of the QT interval. With a sinus rhythm, QT and RR intervals were averaged over three consecutive complexes. During other rhythms, QT and RR intervals were averaged over all complexes on lead II. QTc of ≥500 milliseconds was considered significantly prolonged in both genders; >470 milliseconds in men and >480 milliseconds in women was considered moderately prolonged^6^, ^18^ and represents the 99th percentile for QTc.^19^ Cohen's kappa statistic, for inter‐observer agreement, was made from a subgroup of 200 randomly chosen ECGs evaluated by a third observer. Ethical permission was sought but was considered not necessary by the hospital's Research and Innovation (R&I) department.

### 2.1 Statistical analysis

Normality of distribution was assessed using the Kolmogorov–Smirnov test. Comparison of normally distributed data was made by student's unpaired *t* test assuming equal or unequal variance as necessary. Correlation of heart rate with Bazett formula and to Fridericia formula was with Pearson's correlation. Comparison of length of stay was made with Wilcoxon rank sum test. The proportion of patients surviving their hospital admission, and proportions being readmitted within 1, 3 or 12 months after discharge was made by a 2 × 2 Fisher exact test, with Sidak correction for multiple testing.

A multivariate logistic regression model was used to determine variables relating to the presence/absence of long QTc. The variables decided upon, *a priori*, to be included were: age, gender, potassium, calcium, magnesium concentrations, medication use and presence of diabetes. To examine the influence of long QTc on length of stay, a multiple linear regression model was constructed that included any variable associated with long QTc with *P *<* *0.10. SPSS 24.0 (SPSS Inc, Chicago, IL) was used for statistical analyses. Data are mean ± SD unless stated otherwise. Statistical significance was considered if *P *<* *0.05.

## 3 RESULTS

**Figure 1:**
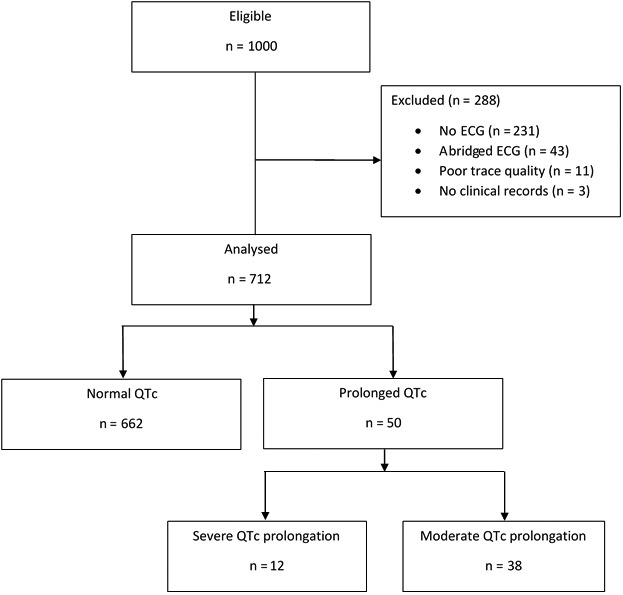
Study flow diagram

Of 1000 consecutive admissions 285 cases were excluded, leaving 712 patients (361 males and 351 females; Figure 1). The reason for admission is provided in Table S1. There were 50 cases of QTc prolongation (31 males and 19 females) giving an incidence of 7%. Of the 50 cases, n = 12 were severe QTc prolongation (≥500 milliseconds), an overall incidence of 1.7%. Of the 50 cases, 18 (36%) had bundle branch block. Of the 662 without long QT, 75 (11.3%) had bundle branch block (*P *<* *0.001).

The automatic QTc measurement recorded a significantly greater proportion of patients with long QTc (n = 111 [15.6%], *P* < 0.001), and severe QTc prolongation (n = 42 [5.9%], *P *<* *0.001) than with manual Bazett. A sensitivity analysis was performed for the comparison of manual vs automatic classification of long QTc by excluding all patients with bundle branch block: 71 (11.5%) patients had long QTc by automatic reader (n = 548 with normal QTc), whereas 32 (5.2%) had long QTc by manual Bazzet (n = 587 with normal QTc; *P *<* *0.001). The heart rate was 87.0 ± 22.2 per minute. QTcF identified only nine patients (1.3%) with prolonged QT, of which three (0.4%) were ≥500 milliseconds. The mean (95% confidence interval) for the difference in QTc between Bazett and Fridericia formulae was 23.1 milliseconds (21.8‐24.3). The Pearson's correlation between heart rate and QTc with Bazett was *r* = 0.157 (*P *<* *0.001) and heart rate to QTcF was *r* = −0.270 (*P *<* *0.001).

Admission potassium was available in n = 581 (82%), calcium in n = 703 (99%), magnesium in n = 603 (85%). The admission potassium and the proportion of patients using QT‐prolonging drugs on admission were significantly different in those patients with prolonged QTc (Table 1).

**Table 1:** Comparison of candidate factors reported to affect QTc

Patient characteristic	Normal QTc n = 662	Long QTc n = 50	*P* value
Age	62.8 ± 19.3	68.1 ± 19.7	0.060
Gender	Male = 330; female = 332	Male = 31; female = 19	0.108
Presence of diabetes mellitus	Yes = 176; no = 486	Yes = 19; no = 31	0.099
Serum potassium at admission (mmol/L)	4.3 ± 0.7	3.9 ± 0.7	<0.001
Serum calcium at admission (mmol/L)	2.25 ± 0.12	2.24 ± 0.13	0.564
Serum magnesium at admission (mmol/L)	0.78 ± 0.11	0.76 ± 0.18	0.554
Presence of QTc‐prolonging drugs at admission	Yes = 294; no = 368	Yes = 35; no = 15	0.009

Long QTc defined as >470 ms males; >480 ms females.

Kappa inter‐observer agreement for long QTc with manual Bazett was 0.65 indicating substantial agreement.

### 3.1 In‐hospital prescription of QTc‐prolonging drugs

At admission, 328 patients (46%) were receiving ≥1 QTc‐prolonging drug of any kind. Of this group, 96 patients were using medication known to cause TdP, 70 were using drugs with a possible risk of TdP, 231 using medications with a conditional risk of TdP and 13 were using medications with a risk in congenital long‐QT syndrome.

**Figure 2:**
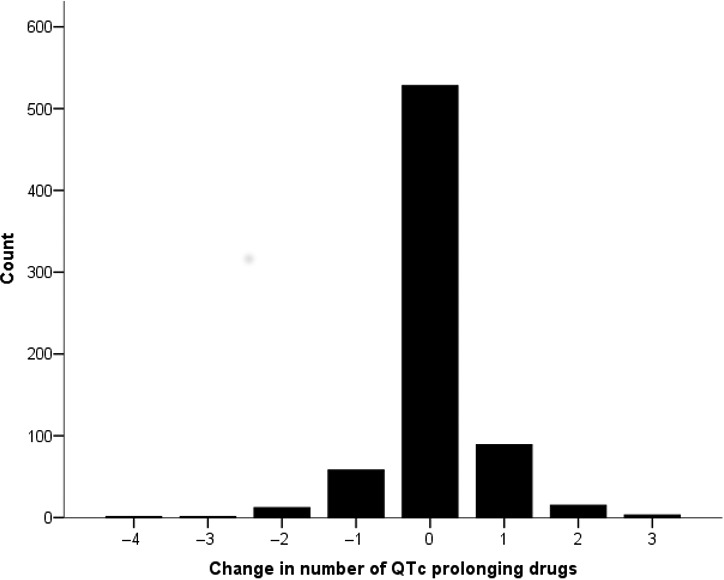
Change in number of medications with potential to prolong QT interval, following admission, in the entire cohort

**Figure 3:**
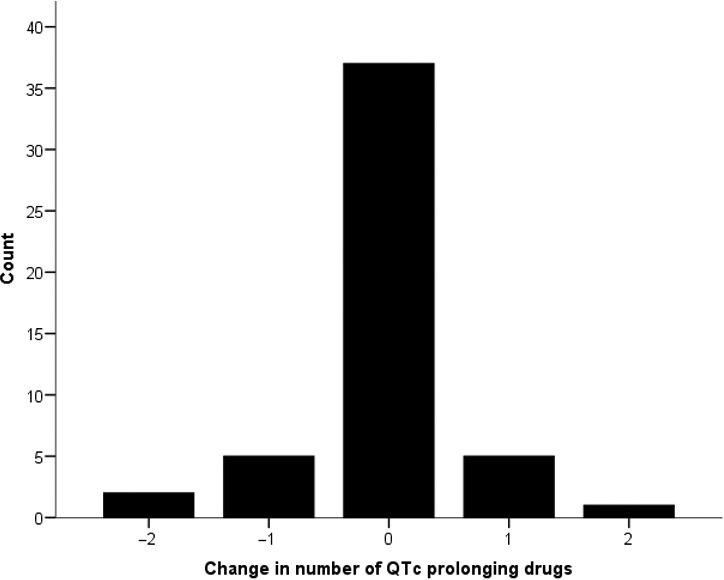
Change in number of medications with potential to prolong QT interval, following admission, in patients with pre‐existing long QTc

Following admission, 512 patients had no change in the number of QTc‐prolonging drugs, 72 had a reduction in number and 105 patients had an increase (Figure 2). Of those with prolonged QTc at admission, seven subsequently had a reduction in number of QT‐prolonging medications, 37 were unchanged and six had an increase (Figure 3).

The QTc‐prolonging drugs observed in this study are shown in Table 2. The mostly frequently prescribed drug (with a known risk of TdP) was Citalopram, although clarithromycin was the most commonly instituted drug in this risk group. Furosemide was the most commonly prescribed drug with any risk of QTc prolongation.

**Table 2:** Prescription of QT‐prolonging drugs following admission

Known risk of torsades de pointes	Possible risk of torsades de pointes	Conditional risk of torsades de pointes	Risk in congenital LQTS
Citalopram n = 34 (+1)	Mirtazapine n = 27 (+1)	Frusemide n = 86 (−8)	Budesonide/Formoterol n = 7 (+2)
Quinine n = 22 (+2)	Risperidone n = 15 (+2)	Amitriptyline n = 28 (−1)	Co‐trimoxazole n = 2 (+2)
Clarithromycin n = 20 (+15)	Ciprofloxacin n = 14 (+8)	Sertraline n = 25 (−3)	Salmeterol n = 1 (−1)
Domperidone n = 11 (+2)	Promethazine n = 10 (0)	Metoclopramide n = 24 (+6)	Terbutaline n = 1 (−1)
Ondansetron n = 11 (+6)	Venlafaxine n = 5 (−1)	Omeprazole n = 15 (+3)	Formoterol n = 0 (−1)
Haloperidol n = 10 (+6)	Buprenorphine n = 4 (+1)	Metronidazole n = 11 (+10)	
Amiodarone n = 9 (+4)	Aripiprazole n = 3 (0)	Fluoxetine n = 9 (−4)	
Donepezil n = 7 (0)	Nortriptyline n = 2 (0)	Indapamide n = 9 (−3)	
Methadone n = 6 (0)	Alfuzosin n = 1 (0)	Olanzapine n = 9 (+4)	
Fluconazole n = 3 (−1)	Atomoxetine n = 1 (+1)	Solifenacin n = 7 (−1)	
Azithromycin n = 2 (0)	Lithium n = 1 (0)	Quetiapine n = 6 (−1)	
Erythromycin n = 2 (−1)	Morphine n = 1 (+1)	Ritonavir n = 5 (0)	
Flecainide n = 1 (0)	Tolterodine n = 1 (0)	Amisulpride n = 4 (0)	
Levomepromazine n = 1 (0)	Vardenafil n = 1 (0)	Bendrofluazide n = 4 (−2)	
Sotalol n = 1 (0)	Clozapine n = 0 (−2)	Ivabradine n = 3 (0)	
	Dasatinib n = 0 (−1)	Hydrochlorothiazide n = 2 (−2)	
	Depixol n = 0 (−1)	Loperamide n = 2 (+1)	
	Efavirenz n = 0 (−1)	Paroxetine n = 2 (0)	
		Chloroquine n = 1 (+1)	
		Posaconazole n = 1 (0)	
		Ranolazine n = 1 (−1)	
		Trazodone n = 1 (−2)	
		Voriconazole n = 1 (+1)	
		Hydroxyzine n = 0 (−2)	

Changes in prescribing are in parentheses.

### 3.2 Patient outcomes

Length of stay was greater in those with prolonged QTc (median 7.2, IQR 2.4‐13.2 days) than in those without (median 3.3, IQR 1.3‐10.0 days; *P* = 0.004). However, in a linear regression model for length of stay, using predictor variables with *P *<* *0.1 (from Table 1), only patient age was significant (β coefficient 0.215; *P *<* *0.001). None of the following affected the length of stay: presence of long QTc (β 0.031; *P *=* *0.457), presence of diabetes (β 0.044; *P* = 0.293), admission potassium (β −0.08; *P *=* *0.058). Twenty‐eight patients died during the index admission (age 84 [IQR 77.5‐88.5] years vs 65 [IQR 49‐79]; *P* < 0.001). Three individuals who died had long QTc, of which one (solid‐organ cancer) was severely prolonged. There were no differences in the proportions of those who survived admission or in the rate of readmission (Table 3).

**Table 3:** Clinical outcomes of long QT

Outcome	Normal QTc N = 662	Long QTc N = 50	*P* value
Length of stay (days); median (IQR)	3.3 (1.3‐10.0)	7.2 (2.4‐13.2)	0.004
In‐hospital mortality	Yes = 25 (3.8%); no = 637	Yes = 3 (6.0%); no = 47	0.438
1‐month readmission (excluding in‐hospital deaths)	Yes = 91; no = 544	Yes = 10; no = 37	0.202
3‐month readmission	Yes = 179; no = 456	Yes = 16; no = 31	0.405
12‐month readmission	Yes = 304; no = 331	Yes = 26; no = 21	0.366

## 4 DISCUSSION

This study has shown that approximately 1 in 14 patients admitted to an acute unit had prolonged QT interval as judged by a manual Bazett method. Ventricular arrhythmias are most often associated with QTc values of 500 milliseconds or more^17^, ^20^ and in this group (severe long QTc) the incidence was 1.7%. This is the first major study reporting incidence in acute medical admissions. By contrast, prevalence data have shown that prolonged QTc is frequently encountered in an acute stroke unit^5^ or CCU.^6^ These data likely reflect the case mix of the various cohorts, as transmural myocardial ischaemia^21^ and intraventricular haemorrhage^22^ are factors associated with long QT, but are usually seen within a high‐dependency environment. However, long QT (particularly drug‐induced long QT) has also been associated with increasing age, congestive cardiac failure, hypokalaemia^4^ and anti‐psychotic medication. Individuals with major psychiatric disorders, with cardiovascular diseases and elderly people comprise a sizeable proportion of the acute medical population. Acutely ill patients are likely to have multiple acquired risk factors for QT prolongation.^23^ Our data suggest that low‐normal plasma potassium has a role in prolonging the QT interval and is a concern given the large number of patients prescribed potassium wasting diuretics. Hypokalaemia (or low‐normal plasma potassium) should be sought and corrected following the initial clinical encounter, especially in those with (or at risk) of QTc prolongation.

Automatic QT interval measurement, integrated into the electrocardiographic device, is convenient for routine use but can be imprecise.^24^ In our series, the automatic measure overestimated the QT interval compared to manual evaluation—even in patients without bundle branch block. Why this occurred is uncertain although discordance has been suggested to relate to difficulties in delineating the end of T wave either due to ‘noise’ or in cases where the T wave is flat, bifid, biphasic, or overlapping on a U wave.^25^ Conversely, the Fridericia formula gave a far lower estimation of incidence of prolonged QT, with a mean difference of 23 milliseconds between Bazett and Fridericia formulae. This difference was larger than previously reported.^26^ Fridericia formula may be more accurate in the context of tachycardia,^17^ although Bazett's formula is thought to adequately correct for heart rates varying between 50 and 90 beats per minute^27^, ^28^; of note the mean heart rate in this study was 87/min and 40% of patients had a pulse over 90/min. The correlation data suggested that QTc tended to shorten with tachycardia using Fridericia, whereas Bazett formula had a positive correlation with heart rate.

The prescribing patterns reflect the case mix seen on an AMU. NICE guideline recommends consideration of dual antibiotic therapy with amoxicillin and a macrolide (such as clarithromycin) for patients with moderate‐ or high‐severity community‐acquired pneumonia.^29^ Use of fluoroquinolones, which have been a cause for concern to their risk for TdP,^30^ are not promoted as first line.^29^ Safety issues have also been raised for use of QTc‐prolonging anti‐psychotic medications.^2^, ^31^ Such drugs are frequently used in‐hospital inpatients as delirium can affect a fifth of acute medical admissions.^32^ NICE guidelines allow for the antipsychotics haloperidol and olanzapine to be used for up to 1‐week as a therapy for delirium.^33^ Whether QTc contributes to increased hospital mortality in those with delirium is unknown.^34^ Prolonged QT interval is known to predict mortality in a variety of other conditions that are frequently encountered in the AMU such as: coronary artery disease,^35^ heart failure^36^ and diabetes mellitus.^37^, ^38^ Survival curves of those with/without prolonged QTc separated well within 50 days of admission, in a hospital‐wide study.^7^ Our data have shown in‐hospital mortality of 3.8% in those with normal QTc and 6.0% of those with long QTc. The study was not powered for this outcome but such a difference would be clinically relevant; based upon these data, approximately 1500 patients would be needed to be studied to detect a difference with 80% power and alpha level of 5%. The more prolonged length of stay (in patients with long QTc) in the unadjusted analysis was unexpected but was no longer significant when correcting for patient age and presence of diabetes or electrolyte disturbance.

The strengths of our study include the robust inter‐observer reproducibility of long QTc diagnosis, the high‐degree of capture of biochemical data, and complete access to electronic drug prescriptions and pharmacy reconciliation with primary care. There are some limitations to our study. First, retrospective cohort studies are susceptible to selection bias although this was minimised using consecutive cases. Second, as discussed, the adequacy of Bazett's formula has been questioned as it may overcorrect the QT interval at fast heart rates and under‐corrects it at low heart rates.^27^, ^28^, ^39^ Third, we do not have data of the prevalence of coronary insufficiency or left ventricular dysfunction; these are risk factors for ventricular arrhythmia in the setting of prolonged QTc.^1^ Finally, this study was designed to determine the incidence of long QTc on presentation to an AMU; therefore the first ECG was evaluated but not any follow‐up ECGs. It may be the case that a patient's long QTc resolved prior to use of QTc‐prolonging drugs. However, prolonged QTc may also develop after admission. In a study of 178 patients prescribed QTc‐prolonging drugs, 26 patients (15%) went on to develop a prolonged QTc within 11‐days of starting a QTc‐prolonging drug.^40^ Finally, we did not find that prolonged QTc independently affected length of stay or in‐hospital mortality. This may represent a type II error as the study was not powered for these outcomes and the number of deaths was relatively low. Data from a HDU/ICU environment have suggested that, in an adjusted model, QT prolongation (captured on continuous bedside monitoring) nearly triples the odds for in‐hospital mortality and prolonged length of stay.^41^

In conclusion, we have shown that QT prolongation, judged by the Bazett formula, is relatively common in acute medical admissions and that low plasma potassium (albeit within the normal range) and QT‐prolonging drugs contribute to this. The incidence of prolonged QTc varied considerably by the formula used for heart rate correction. Whether prolonged QTc interval affects clinical outcomes, in this group of patients, requires further study.

## DISCLOSURE

R.M, A.G., A.N. and M.B.W. no conflict of interest.

## AUTHOR CONTRIBUTIONS

R.M. and A.G. collected the clinical data and ECG analysis. A.N. independently reviewed the ECG data. R.M, A.G and M.B.W. analysed all the data and wrote the manuscript. All authors reviewed the manuscript. M.B.W is the guarantor of this work and, as such, had full access to all the data in the study and takes responsibility for the integrity of the data and the accuracy of the data analysis.

## References

[1] Drew BJ , Ackerman MJ , Funk M , et al. Prevention of torsade de pointes in hospital settings: a scientific statement from the American Heart Association and the American College of Cardiology Foundation. J Am Coll Cardiol. 2010;55(9):934‐947. 20185054 10.1016/j.jacc.2010.01.001 PMC3057430

[2] Rabkin SW . Aging effects on QT interval: implications for cardiac safety of antipsychotic drugs. J Geriatr Cardiol. 2014;11(1):20‐25. 24748877 10.3969/j.issn.1671-5411.2014.01.005 PMC3981979

[3] Reardon M , Malik M . QT interval change with age in an overtly healthy older population. Clin Cardiol. 1996;19(12):949‐952. 8957599 10.1002/clc.4960191209

[4] Roden DM . Drug‐induced prolongation of the QT interval. N Engl J Med. 2004;350(10):1013‐1022. 14999113 10.1056/NEJMra032426

[5] Goldstein DS . The electrocardiogram in stroke: relationship to pathophysiological type and comparison with prior tracings. Stroke. 1979;10(3):253‐259. 462510 10.1161/01.str.10.3.253

[6] Tisdale JE , Wroblewski HA , Overholser BR , et al. Prevalence of QT interval prolongation in patients admitted to cardiac care units and frequency of subsequent administration of QT interval‐prolonging drugs: a prospective, observational study in a large urban academic medical center in the US. Drug Saf. 2012;35(6):459‐470. 22612851 10.2165/11598160-000000000-00000

[7] Haugaa KH , Bos JM , Tarrell RF , et al. Institution‐wide QT alert system identifies patients with a high risk of mortality. Mayo Clin Proc. 2013;88(4):315‐325. 23541006 10.1016/j.mayocp.2013.01.013

[8] Bogossian H , Frommeyer G , Ninios I , et al. A new experimentally validated formula to calculate the QT interval in the presence of left bundle branch block holds true in the clinical setting. Ann Noninvasive Electrocardiol. 2017;22(2):e12393. 10.1111/anec.12393 PMC6931597 27562181

[9] Crow RS , Hannan PJ , Folsom AR . Prognostic significance of corrected QT and corrected JT interval for incident coronary heart disease in a general population sample stratified by presence or absence of wide QRS complex: the ARIC Study with 13 years of follow‐up. Circulation. 2003;108(16):1985‐1989. 14517173 10.1161/01.CIR.0000095027.28753.9D

[10] Fukui S , Katoh H , Tsuzuki N , et al. Multivariate analysis of risk factors for QT prolongation following subarachnoid hemorrhage. Crit Care. 2003;7(3):R7‐R12. 12793884 10.1186/cc2160 PMC270671

[11] Makkar RR , Fromm BS , Steinman RT , et al. Female gender as a risk factor for torsades de pointes associated with cardiovascular drugs. JAMA. 1993;270(21):2590‐2597. 8230644 10.1001/jama.270.21.2590

[12] Gill GV , Woodward A , Casson IF , et al. Cardiac arrhythmia and nocturnal hypoglycaemia in type 1 diabetes–the ‘dead in bed’ syndrome revisited. Diabetologia. 2009;52(1):42‐45. 18972096 10.1007/s00125-008-1177-7

[13] Trojak B , Astruc K , Pinoit JM , et al. Hypokalemia is associated with lengthening of QT interval in psychiatric patients on admission. Psychiatry Res. 2009;169(3):257‐260. 19747736 10.1016/j.psychres.2008.06.031

[14] Newman DB , Fidahussein SS , Kashiwagi DT , et al. Reversible cardiac dysfunction associated with hypocalcemia: a systematic review and meta‐analysis of individual patient data. Heart Fail Rev. 2014;19(2):199‐205. 23355181 10.1007/s10741-013-9371-1

[15] Woosley R , Heise CW , Romero KA . www.CredibleMeds.org, QTdrugs List, AZCERT, Inc. 1822 Innovation Park Dr., Oro Valley, AZ 85755

[16] Bazzett H . An analysis of time‐relations of electrocardiograms. Heart. 1920;7:353‐370.

[17] Goldenberg I , Mathew J , Moss AJ , et al. Corrected QT variability in serial electrocardiograms in long QT syndrome: the importance of the maximum corrected QT for risk stratification. J Am Coll Cardiol. 2006;48(5):1047‐1052. 16949500 10.1016/j.jacc.2006.06.033

[18] Shen WK , Sheldon RS , Benditt DG , et al. 2017 ACC/AHA/HRS Guideline for the Evaluation and Management of Patients with Syncope: executive Summary: a Report of the American College of Cardiology/American Heart Association Task Force on Clinical Practice Guidelines and the Heart Rhythm Society. J Am Coll Cardiol. 2017;70(5):620‐663. 28286222 10.1016/j.jacc.2017.03.002

[19] Taggart NW , Haglund CM , Tester DJ , et al. Diagnostic miscues in congenital long‐QT syndrome. Circulation. 2007;115(20):2613‐2620. 17502575 10.1161/CIRCULATIONAHA.106.661082

[20] Priori SG , Schwartz PJ , Napolitano C , et al. Risk stratification in the long‐QT syndrome. N Engl J Med. 2003;348(19):1866‐1874. 12736279 10.1056/NEJMoa022147

[21] Kenigsberg DN , Khanal S , Kowalski M , et al. Prolongation of the QTc interval is seen uniformly during early transmural ischemia. J Am Coll Cardiol. 2007;49(12):1299‐1305. 17394962 10.1016/j.jacc.2006.11.035

[22] van Bree MD , Roos YB , van der Bilt IA , et al. Prevalence and characterization of ECG abnormalities after intracerebral hemorrhage. Neurocrit Care. 2010;12(1):50‐55. 19813104 10.1007/s12028-009-9283-z

[23] Zeltser D , Justo D , Halkin A , et al. Torsade de pointes due to noncardiac drugs: most patients have easily identifiable risk factors. Medicine (Baltimore). 2003;82(4):282‐290. 12861106 10.1097/01.md.0000085057.63483.9b

[24] Malik M . Errors and misconceptions in ECG measurement used for the detection of drug induced QT interval prolongation. J Electrocardiol. 2004;37(Suppl):25‐33. 10.1016/j.jelectrocard.2004.08.005 15534789

[25] Sano M , Aizawa Y , Katsumata Y , et al. Evaluation of differences in automated QT/QTc measurements between Fukuda Denshi and Nihon Koden systems. PLoS ONE. 2014;9(9):e106947. 25229724 10.1371/journal.pone.0106947 PMC4167700

[26] Vandenberk B , Vandael E , Robyns T , et al. Which QT correction formulae to use for QT monitoring? J Am Heart Assoc. 2016;5(6):e003264. 10.1161/JAHA.116.003264 PMC4937268 27317349

[27] Hnatkova K , Malik M . “Optimum” formulae for heart rate correction of the QT interval. Pacing Clin Electrophysiol. 1999;22(11):1683‐1687. 10598974 10.1111/j.1540-8159.1999.tb00390.x

[28] Moss AJ , Long QT . Syndrome. JAMA. 2003;289(16):2041‐2044. 12709446 10.1001/jama.289.16.2041

[29] National Institute for Health and Care Excellence (NICE) . Clinical guideline [CG191]. Pneumonia in adults: diagnosis and management 2014. 31841289

[30] Stahlmann R , Lode H . Safety considerations of fluoroquinolones in the elderly: an update. Drugs Aging. 2010;27(3):193‐209. 20210367 10.2165/11531490-000000000-00000

[31] Stollberger C , Huber JO , Finsterer J . Antipsychotic drugs and QT prolongation. Int Clin Psychopharmacol. 2005;20(5):243‐251. 16096514 10.1097/01.yic.0000166405.49473.70

[32] Pendlebury ST , Lovett NG , Smith SC , et al. Observational, longitudinal study of delirium in consecutive unselected acute medical admissions: age‐specific rates and associated factors, mortality and re‐admission. BMJ Open. 2015;5(11):e007808. 10.1136/bmjopen-2015-007808 PMC4654280 26576806

[33] National Institute for Health and Care Excellence (NICE) . Clinical guideline [CG103]: Delirium: prevention, diagnosis and management, 2010. 31846262

[34] Salluh JI , Wang H , Schneider EB , et al. Outcome of delirium in critically ill patients: systematic review and meta‐analysis. BMJ. 2015;350:h2538. 26041151 10.1136/bmj.h2538 PMC4454920

[35] Chugh SS , Reinier K , Singh T , et al. Determinants of prolonged QT interval and their contribution to sudden death risk in coronary artery disease: the Oregon Sudden Unexpected Death Study. Circulation. 2009;119(5):663‐670. 19171855 10.1161/CIRCULATIONAHA.108.797035 PMC2734945

[36] Vrtovec B , Delgado R , Zewail A , et al. Prolonged QTc interval and high B‐type natriuretic peptide levels together predict mortality in patients with advanced heart failure. Circulation. 2003;107(13):1764‐1769. 12665499 10.1161/01.CIR.0000057980.84624.95

[37] Cox AJ , Azeem A , Yeboah J , et al. Heart rate‐corrected QT interval is an independent predictor of all‐cause and cardiovascular mortality in individuals with type 2 diabetes: the Diabetes Heart Study. Diabetes Care. 2014;37(5):1454‐1461. 24574343 10.2337/dc13-1257 PMC4182905

[38] Rossing P , Breum L , Major‐Pedersen A , et al. Prolonged QTc interval predicts mortality in patients with Type 1 diabetes mellitus. Diabet Med. 2001;18(3):199‐205. 11318840 10.1046/j.1464-5491.2001.00446.x

[39] Karjalainen J , Viitasalo M , Manttari M , et al. Relation between QT intervals and heart rates from 40 to 120 beats/min in rest electrocardiograms of men and a simple method to adjust QT interval values. J Am Coll Cardiol. 1994;23(7):1547‐1553. 8195512 10.1016/0735-1097(94)90654-8

[40] Vandael E , Vandenberk B , Vandenberghe J , et al. Development of a risk score for QTc‐prolongation: the RISQ‐PATH study. Int J Clin Pharm. 2017;39(2):424‐432. 28281228 10.1007/s11096-017-0446-2

[41] Pickham D , Helfenbein E , Shinn JA , et al. High prevalence of corrected QT interval prolongation in acutely ill patients is associated with mortality: results of the QT in Practice (QTIP) Study. Crit Care Med. 2012;40(2):394‐399. 22001585 10.1097/CCM.0b013e318232db4a

