# Supplementary material for: Incidence and outcomes of long QTc in acute medical admissions
Source: Int J Clin Pract. 2018 Sep 17;72(11):e13250. doi: 10.1111/ijcp.13250 (PMC6220840; doi:10.1111/ijcp.13250)
Supplement: Supplementary file 1 [file IJCP-72-na-s001.docx]

| **System** | **Count** |
| --- | --- |
| Cardiology | 191 |
| Dermatology | 28 |
| Drug reaction | 1 |
| Endocrine | 51 |
| Ear, Nose & Throat (ENT) | 3 |
| Gastroenterology | 119 |
| Gynaecology | 1 |
| Haematology | 65 |
| Musculoskeletal | 24 |
| Neurology | 109 |
| Oncology | 9 |
| Psychiatry | 50 |
| Renal | 100 |
| Respiratory | 207 |
| Rheumatology | 7 |
| Other | 35 |

**Supplementary Table 1**: Reason for admission (by system) of study cohort (n=1000)
